# Is canine calprotectin in serum stabile after storage at low temperature?

**DOI:** 10.1186/s12917-022-03534-8

**Published:** 2022-12-24

**Authors:** Tara Kostanjšak, Krunoslav Bojanić, Helena Čičak, Jelena Gotić, Zoran Vrbanac, Ana-Maria Šimundić, Dražen Vnuk, Nika Brkljača Bottegaro

**Affiliations:** 1 Clinic for surgery, orthopaedics and ophthalmology, Faculty of Veterinary medicine, University of Zagreb, Zagreb, Croatia; 2 Laboratory for Aquaculture Biotechnology, Division of Materials Chemistry, Ruđer Bošković Institute, Zagreb, Croatia; 3 Department of Medical Laboratory Diagnostics, University Hospital “Sveti Duh”, Zagreb, Croatia; 4 Clinic for Internal Medicine, Faculty of Veterinary Medicine, University of Zagreb, Zagreb, Croatia; 5 Department of Radiology, Ultrasound Diagnostic and Physical Therapy, Faculty of Veterinary Medicine, University of Zagreb, Zagreb, Croatia

**Keywords:** Calprotectin, Serum, Stability, Imprecision, Canine

## Abstract

**Background:**

In human and veterinary medicine calprotectin is most widely used in diagnosing different gastro-intestinal diseases. The aim of this study was to assess the stability of canine calprotectin (cCP) in serum after storage at low temperatures and imprecision of the method.

**Methods:**

Blood samples were collected from dogs with different clinical diagnoses. Twenty-two dogs were included in this study. Calprotectin concentration was measured 4 hours after serum separation (T0), and after being frozen at − 80 °C for 8 (T1) and 16 weeks (T2). The maximum permissible difference (MPD) was derived from the equation for calculating total error (TE) TE = %Bias + (1.96 x %CV), where bias and coefficient of variation (CV) were defined by the manufacturer. The dogs enrolled in this study were patients admitted during the morning (9–12 a.m.), on the day the first measurement was performed. All sample analysis for determination of stability were done in duplicates. For determination of within-run precision, the two patients’ serum samples were analyzed in 20 replicates. Imprecision was assessed by analyzing 20 replicates on one plate on two samples where high and low concentrations were anticipated.

**Results:**

The calculated value of MPD was 32.52%. Median calprotectin concentrations were higher at T1 114.08 μg/L (IQR = 55.05–254.56) and T2 133.6 μg/L (IQR = 100.57–332.98) than at T0 83.60 μg/L (IQR = 50.38–176.07). Relative and absolute bias at T1 (49.3%; 45.98 μg/L) and T2 (109.93%; 94.09 μg /L) have shown that calprotectin concentrations increase after long term storage at − 80 °C.

**Conclusion:**

The results of the present study indicate that c-CP was not stable for 16 weeks at low storage temperature (− 80 °C). Considering the observed change in the concentration of c-CP at T1, a storage time of 8 weeks should be safely applied. The method imprecision was not satisfactory, especially in the lower concentration range.

## Background

Acute-phase proteins are considered to be an integral part of the early inflammatory response that occurs due to infection, trauma, stress or neoplastic changes. In addition to inflammatory conditions acute phase proteins are also released in physiological conditions such as pregnancy [1]. Calprotectin is an acute-phase protein recognized as a promising biomarker of both acute and chronic inflammation [2] that belongs to the group of S100 leukocyte proteins primarily secreted by neutrophilic granulocytes and monocytes. The calprotectin heterocomplex consists of two different subunits, the light subunit, S100A8 (also known as calgranulin A or MRP8), and the heavy subunit, S100A9 (also known as calgranulin B or MRP14) [3]. An important ability of calprotectin is its potential to bind calcium and zinc [4] giving calprotectin antibacterial properties and affecting its stability. Moreover, zinc-binding domains allow calprotectin to have antibacterial activity [5] by permeating into the cell membrane of microorganisms and inhibiting their growth [6, 7]. Recently, calprotectin has been described as a potential endogenous activator of Toll-like receptor 4 (TLR-4) that induces endotoxic shock [8]. The presence of calcium induces conformational changes in the heterodimer, thereby allowing binding of other proteins. Calcium ions stabilize calprotectin structure and also help to keep its structure upon chelation of metal ions which is involved in defense against microorganisms [9]. Calprotectin is an important mediator of many regulatory functions such as chemotactic activity, macrophage deactivation, and inhibition of immunoglobulin synthesis [10]. In the presence of calcium, calprotectin is resistant to proteolytic degradation and this is what forms the basis of its stability in feces [11].

Serume calprotectine correlates strongly with fecal calprotectin in patients with inflammatory bowel disease (IBD). It is possible to measure calprotectin concentration from feces and serum but both methods have their advantages and disadvantages. For example, taking a feces sample is non-invasive, but on the other hand, the serum is more accessible, especially in emergency and critical patients because blood is drawn for analysis of other blood parameters. The advantage of stool samples is that feces is in direct contact with the intestinal mucosa and reflects inflammatory processes localized in the gastro-intestinal tract best. Serum calprotectin is a systemic indicator of inflammation and its interpretation is possible the same way as c-CRP. A disadvantage of the evaluation of fecal calprotectin is inhomogenous fecal sample, which can have higher or lower calprotectin concentration than mean calprotectin concentration in feces. A disadvantage of the evaluation of serum calprotectin is its instability at room temperature (4 days) [3] as well as the need for cooling devices with temperatures as low as − 80 °C. In human and veterinary medicine serume calprotectin is most widely used in diagnosing IBD, as effective inflammatory marker [12, 13], and rheumatoid diseases [14]. The several studies observed increased calprotectin serum concentration in septic patient. Larsson et al. [15] proved that calprotectin in plasma is superior to procalcitonin in predicting 30-day mortality in intensive care unit patients and that it is superior in distinguishing non-sepsis from sepsis patients. In neonatal human babies’ calprotectin is considered as a promising, sensitive and specific biomarker of sepsis [16]. Serum calprotectin has been described as a prognostic factor for dogs with sepsis and systemic inflammatory response syndrome (SIRS) [17].

While the stability of human calprotectin has been investigated extensively, to the present moment, no studies regarding the stability of canine calprotectin (c-CP) in serum stored at − 80 °C for 16 weeks have been published. Haisma et al. [18] in their study noted that calprotectin in stool is not stable at room temperature but that its stability increases while kept at lower temperatures (4 °C) for 6 days.

The aim of the present study was to determine the stability of c-CP in serum and imprecision of method used to measure it. This study aimed to assess the stability of calprotectin to ensure correct storage time duration at low temperature at which calprotectin is stable and to estimate imprecision of ELISA for measurement of calprotectin concentrations in canine serum. The hypothesis of this study was that serum c-CP concentration depends on the duration of storage.

## Results

The median age of the 22 dogs enrolled in this study was 7,12 ± 3,75 years (mean ± SD). The youngest dog had 5 months while the oldest animal had 13 years. There were 8 different dog breeds while the most represented one was mixed breed (*n* = 5). Fourteen dogs were males while 8 were females. Two dogs were used for the imprecision test and 17 dogs for the measurement of serum c-CP. Three dogs were excluded from the analysis due to highly lipemic and hemolytic serum samples. Hemolytic samples were excluded. Serum from two dogs was used only to measure imprecision.

Average values of c-CP concentrations in dog serum measured in 17 dogs in three time points are shown in Table 1.

**Table 1:** Medians and ranges of calprotectin concentrations measured in canine serum at T0, T1 and T2 from 17 dogs analysed in duplicates

*Calprotectin,* μg*/L*	*T0*	*T1*	*T2*
*Range (μg/L)* *Median (IQR)*	18.10–366.96 83.60 (50.38–176.07)	14.14–531.88 114.08 (55.05–254.56)	59.58–623.39 133.61 (100.57–332.98)

Calprotectin concentrations are presented as median and interquartile range (IQR). *T0* calprotectin, μg*/L* baseline calprotectin serum concentrations; *T1* calprotectin, *μg/L* calprotectin serum concentrations measured after 8 weeks of storage; *T2* calprotectin, *μg/L* calprotectin serum concentration measured after 16 weeks

Relative calprotectin bias at T1 was 49.3% and absolute bias was 45.98 μg/L. At T2 relative bias was 109.93% and absolute bias was 94.09 μg/L. Total analytical error was calculated by Eq. 2. and amounted to 32.52. Percentage difference was calculated for 17 serum samples and stability is shown in Table 2. as PD% from average baseline value at T0 in two time point (T1 and T2).

**Table 2:** Stability of calprotectin in canine serum stored at − 80 °C

Test	Number of participants	T0	T1	T2	Instability Equation
Calprotectin (μg/L)	17	128.38 (18.1–366.96)	3.75% (−36.17%, 39.85%)	40.61% (27.10%, 55.07%)	PD% = 2.124 x time (weeks) *P* = 0.008 R^2^ = 0.191

Results for time points T1 and T2 are shown as PD%* (95% CI**)

*PD% percentage difference (%), **CI confidence interval

**Fig. 1:**
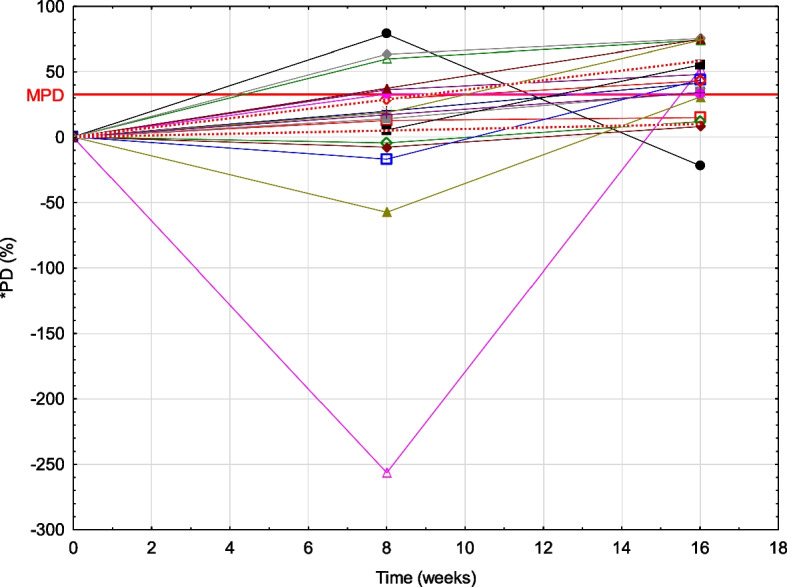
Stability of canine serum calprotectin concentrations at −80 °C. Instability equation calculation using the least squares adjustment with confidence intervals (dotted lines for the slope). All patient data is also shown. Red line presents the MPD (maximum permissible difference) for serum calprotectin (which was 32.52% at the time). *PD (%) - percentage difference (%)

**Fig. 2:**
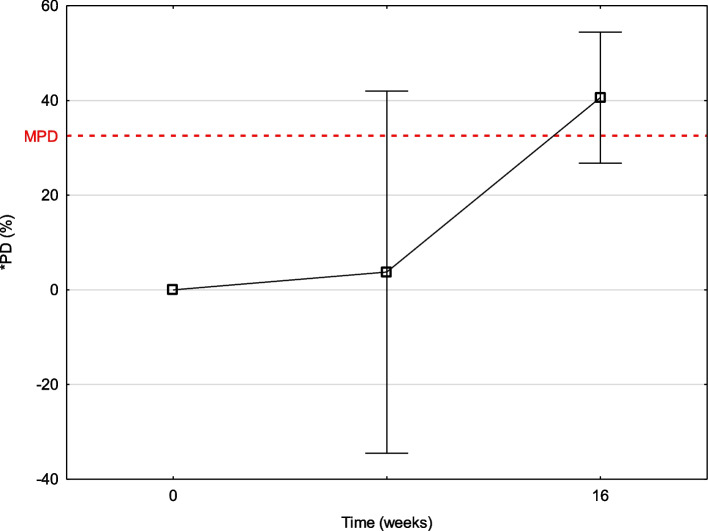
Stability equation calculation using the point-to-point estimation with confidence intervals for the mean of patients at every study time. Red line presents the MPD (maximum permissible difference) for serum calprotectin (which was 32.52% at the time). *PD (%) - percentage difference (%)

Average calprotectin concentrations in three time points and PD (%) for 17 dogs at each time point is shown in Table 3. There was a significant increase in calprotectin concentration in samples stored for 8 and 16 weeks in comparison to the baseline values (Figs. 1 and 2).

**Table 3:** Average calprotectin concentrations in three time points and PD (%) for individual participants at each time point demonstrating the stability of c-CP in serum at low temperature (− 80 °C) in closed Eppendorf tubes (Nuova Aptaca, Italy)

(Calprotectin μg/L)		Basal	8 weeks	16 weeks
Dog 1	Average	176.07	201.62	206.81
	PD% from basal		12.67%	14.86%
Dog 2	Average	366.96	351.27	415.94
	PD% from basal		−4.47%	11.78%
Dog 3	Average	50.38	14.14	104.89
	PD% from basal		− 256.37%	51.97%
Dog 4	Average	114.71	531.88	94.06
	PD% from basal		78.43%	− 21.95%
Dog 5	Average	219.06	254.56	332.98
	PD% from basal		13.95%	34.21%
Dog 6	Average	93.46	86.71	101.61
	PD% from basal		−7.78%	8.02%
Dog 7	Average	41.30	26.22	59.58
	PD% from basal		−57.52%	30.68%
Dog 8	Average	72.87	114.08	139.53
	PD% from basal		36.13%	47.78%
Dog 9	Average	78.45	97.76	133.61
	PD% from basal		19.75%	41.28%
Dog 10	Average	64.38	55.05	114.34
	PD% from basal		−16.94%	43.70%
Dog 11	Average	83.60	117.69	146.44
	PD% from basal		28.96%	42.91%
Dog 12	Average	18.10	44.84	69.12
	PD% from basal		59.63%	73.81%
Dog 13	Average	328.25	489.09	488.51
	PD% from basal		32.89%	32.81%
Dog 14	Average	279.90	295.75	623.39
	PD% from basal		5.36%	55.10%
Dog 15	Average	25.04	68.02	100.57
	PD% from basal		63.19%	75.10%
Dog 16	Average	18.28	29.14	71.58
	PD% from basal		37.27%	74.46%
Dog 17	Average	151.68	186.24	579.02
	PD% from basal		18.56%	73.80%
Average PD%*			3.75%	40.61%
CI 95%**			(−36.17, 39.85%)	(27.10, 55.07%)

*PD% percentage difference (%), **CI confidence interval (%)

The determined CV% for precision did not meet the manufacturer criteria. Calculated imprecision is shown in Table 4.

**Table 4:** Imprecision measured on one plate on two levels of canine serum calprotectin expressed as CV%

Calprotectin concentration (μg/L)	CV%*	Manufacturer declaration
Low serum concentration	26.4%	< 10%
High serum concentration	32.9%	< 10%

*CV% coefficient of variation (%)

## Discussion

The results showed that serum calprotectin was not stable after more than 8 weeks of storage at − 80 °C. The concentration of calprotectin was 10 times higher after 16 weeks than the concentration measured after 8 weeks. Reporting of stability was done in accordance with the CRESS guidelines Moreover, the CRESS guidelines stated that the stability of a certain analyte should be determined on a minimum of 10 different samples [19].

Hemolytic samples were excluded because hemolysis can non-specifically elevate calprotectin levels in serum [20] and hyperlipidemia and hypertriglyceridemia were associated with increased calprotectin concentrations in miniature schnauzers [21].

Heilman et al. [22] measured reference interval on 137 dogs that represented healthy control group. Serum calprotectin values ranged from 28.0 to 695.8 μg/L. Calculated reference interval of calprotectin in canine serum was 76.4–536.6 μg/L. Mylemans et al. [23] have determined the stability of calprotectin in serum and plasma at different storage conditions and time points in humans. They concluded that calprotectin values remained stable for up to 7 days at − 20 °C and could endure 5 freeze-thaw cycles. At room-temperature calprotectin was noted to be stable for up to 24 and 72 hours. This study tested stability of canine serume calprotectin for the first time at − 80 °C after 8 and 16 weeks. Pedersen et al. [24] noted that there is a temperature-dependent increase in calprotectin concentrations in human blood. Concentrations of calprotectin increased more in serum samples that were stored at higher temperatures (20 °C and 37 °C) than in samples stored at 4 °C. One of the key findings in their study was the significant impact of storage temperature on samples that were not centrifuged which was in correlation with the calprotectin concentrations and leukocyte and neutrophil count. Therefore, if the serum is used to measure calprotectin concentrations, pre-centrifugation time should be less than 24–48 hours after sampling. The reason for the increase of concentration of calprotectin is theoretically expected because of the degradation of blood cells and due to releasing calprotectin which is contained in leukocytes cytoplasm. In this study, the calprotectin concentration was significantly increased after 16 weeks of storage at -80 °C.

In contrast to the mentioned studies, the temperature at which our serum samples were stored was − 80 °C and the duration of storage was longer. This could be the reason for the difference in stability of calprotectin concentrations. All serum samples were centrifuged in a relatively short time (within 3 hours) and there were no freeze-thaw cycles that could affect calprotectin concentrations in serum.

By assessing 20 replicates on two levels of calprotectin, CV (%) indicated that imprecision was greater than the manufacturer declared for within-run precision by 17% in serum from the dog with gastric dilation/volvulus and by 24% in the dog without GI symptoms. The obtained results suggest that within-run imprecision was greater in the serum with lower levels of calprotectin. Imprecision of method used was not satisfactory. The reason for unsatisfactory imprecision could be manual pipetting of aliquots which could be avoided by using automatic pipetting machine.

In the present time, calprotectin is most often measured from feces samples due to its higher stability. One of the reasons calprotectin is more stable in feces samples than in serum is a higher concentration of calcium which by binding to calprotectin increases its stability making it resistant to proteolytic degradation [3]. An increase in calprotectin concentrations depends on which components the chosen method measures and on the location of epitopes. Unfortunately, in this study, there was no sufficient data provided by the manufacturer.

The present study had some limitations. The within-run precision was verified by analysing 20 replicates on one ELISA plate [25]. Due to the restricted number of ELISA plates that were available for this study, the newer approach which contains 5 replicates by 5 days consecutive and incorporates within- and between- run precision could not be used to verify precision. We only examined the impact of low temperatures during the storage period of 8 and 16 weeks on c-CP concentrations, which does not allow general conclusions to be drawn regarding temperature and time effects on c-CP measurements. However, this does not conflict with the aim of the study, which was the determination of stability of c-CP concentrations in serum. Further research of calprotectin concentrations after different storage times and at different temperatures is necessary to research option that dimers and multimeric forms decompose and that the concentration of calprotectin increases as a result and to define storage conditions most suitable for this molecule.

## Conclusion

To conclude, this study data revealed that c-CP is not stable at − 80 °C in serum samples when stored longer than 8 weeks. Longer storage of serum samples for measurement of calprotectin concentration leads to unacceptable deviations from the true value. The detected imprecision was greater than the manufacturer declared for within-run precision by 17% in the dog with GI symptoms and by 24% in the dog without GI symptoms.

## Methods

All procedures performed in this study involving animals were in accordance with the ethical standards of the Animal Ethics Committee of Faculty of Veterinary Medicine, University of Zagreb, approval no: 640–01/20–02/09; 251–61-01/139–20-27.

### Animals

The dogs enrolled in this study were patients admitted during the morning (9–12 a.m.), on the day the first measurement was performed, at the University Veterinary Hospital at Faculty of Veterinary Medicine in Zagreb. All dogs were chosen randomly and the owners gave a written consent for participation in the study. The dogs were admitted due to various reasons that were not known to the examiners at the time of sampling. History data, physical examination findings, complete blood count and biochemical analysis were noted for all enrolled animals to determine their health status. The analysis of samples was performed in July, September and October 2021.

### Sampling

Blood from each dog was collected on the same day in span of 3 hour, after admission, from the cephalic vein into 3 biochemistry 4 mL tubes with clot activator (LT BURNIK d.o.o., Vodice, Slovenia). The samples were left at room temperature until a clot formed after which the serum was separated by centrifugation for 10 minutes at 3500 x g. Immediately after centrifugation the serum was separated into 5 aliquots (1 ml) in micro test tubes. Aliquot’s analysis to determine c-CP concentration was done at T0 (4 hours after serum centrifugation), at T1 (after 8 weeks of storage) and at T2 (after 16 weeks of storage). Serum samples that were analyzed at T1 and T2 were frozen at − 80 °C (±2 °C) upon centrifugation. Prior to being analyzed samples at T1 and T2 were thawed at room temperature (21 °C). All samples were subjected to homogenization by a vortex.

### Analysis

Serum concentrations of calprotectin were determined with sandwich enzyme-linked immunosorbent assay (ELISA) for in vitro quantitative measurement of canine calprotectin concentration in serum, plasma and other biological fluids – Canine Calprotectin ELISA Kit CALPRO, RK00520 (AbClonal technology, Woburn, USA). The ELISA assay was done manually according to recommended procedure defined by the manufacturer. The negative and positive controls were included in this ELISA kit. All measurements were done on the same lot of reagents.

All sample analysis for determination of stability were done in duplicates. For determination of within-run precision, the two patients’ serum samples were analyzed in 20 replicates. Imprecision was assessed by analyzing 20 replicates on one plate on two levels of calprotectin: one sample (anticipated low level of calprotectin) from a dog without GI symptoms and one sample (anticipated high level of calprotectin) from a dog with gastric dilation/volvulus in which high concentration of calprotectin was anticipated. The two dogs were chosen amongst 22 animals. Coefficient of variation (CV) was calculated as follow:

1 $$\begin{array}{c}\text{CV}\;\left(\%\right)=\left(\text{*SD}/\text{mean}\right)\;\text{x}\;100\\\ast SD\;-\;standard\;deviation\end{array}$$

Manufacturer has declared CV < 10% for the within-run Precision (estimated on 20 replicates at low, middle and high level of calprotectin, on one plate). In this study, it has been used 10% as a performance criterion for imprecision.

The stability was expressed as a percentage difference (PD%) between the baseline results (initial measurement) and results measured at T1 and T2 and was calculated by equation (Eq. 2), for each time point:

2 $$\textrm{PD}\%=\left(\textrm{T}0\hbox{-} \textrm{TX}\right)/\textrm{T}0\ \textrm{x}\ 100$$

Deviation from the baseline value was expressed as absolute (μg/L) and relative (%) bias.

Total error (TE) was used to define performance criteria for stability. Calculation of TE was done by Eq. 3:

3 $$\textrm{TE}=\%\,\textrm{Bias}+\left(1.96\ \textrm{x}\,\%\,\textrm{CV}\right)$$

The manufacturer did not declare any data about calprotectin stability. Declared accuracy (bias) for serum sample was 9% (manufacturer data).

### Statistics

Shapiro-Wilk test was used to test normality of distribution on all data obtained by measuring c-CP with ELISA. All data were distributed normally, and the results were presented as range and median with interquartile range (IQR).

Statistical and exploratory data analyses were performed using R software v.4.0.4 R Core Team (2019). R: A language and environment for statistical computing (R Foundation for Statistical Computing, Vienna, Austria. http://www.R-project.org/), TIBCO Statistica® 13.3.0 (TIBCO Software Inc., Palo Alto, USA; 2017), Microsoft Excel (Microsoft Corporation, Washington, USA, 2019) and MedCalc® Statistical Software version 20.026 (MedCalc Software Ltd., Ostend, Belgium; https://www.medcalc.org; 2022).

## Data Availability

The datasets used and/or analysed during the current study are available from the corresponding author on reasonable request.
